# Trends in incidence and long-term prognosis of acute kidney injury following coronary angiography in Chinese cohort with 11,943 patients from 2013 to 2017: an observational study

**DOI:** 10.1186/s12882-021-02427-6

**Published:** 2021-06-25

**Authors:** Jin Liu, Qiang Li, Disheng Lai, Guoqin Chen, Bo Wang, Liwei Liu, Haozhang Huang, Zhubin Lun, Ming Ying, Guanzhong Chen, Zhidong Huang, Danyuan Xu, Liangguang Meng, Xiaoming Yan, Weiyan Qiu, Ning Tan, Jiyan Chen, Yong Liu, Shiqun Chen

**Affiliations:** 1 Department of Cardiology, Guangdong Provincial Key Laboratory of Coronary Heart Disease Prevention, Guangdong Provincial People’s Hospital, Guangdong Cardiovascular Institute, Guangdong Academy of Medical Sciences, South China University of Technology, 510080 Guangzhou, China; 2 The Second School of Clinical Medicine, Southern Medical University, 510515 Guangzhou, China; 3 Department of Cardiology, Dongguan TCM Hospital, 523209 Dongguan, China; 4 Guangdong Provincial People’s Hospital, School of Medicine, South China University of Technology, 510100 Guangzhou, China; 5 Department of Information Technology, Guangdong Provincial People’s Hospital, Guangdong Academy of Medical Sciences, 510080 Guangzhou, China; 6 Department of Cardiology, Guangzhou Panyu Central Hospital, Guangzhou, China

**Keywords:** Contrast-associated acute kidney injury, Incidence, Trends, Mortality

## Abstract

**Background:**

Contrast-associated acute kidney injury (CA-AKI) is a common complication with poor prognosis after coronary angiography (CAG). With the prevention methods widely being implemented, the temporal trends of incidence and mortality of CA-AKI are still unknown over the last five years. The study aims to determine the incidence and prognosis of CA-AKI in China.

**Methods:**

This retrospective cohort study was based on the registry at Guangdong Provincial People’s Hospital in China (ClinicalTrials.gov NCT04407936). We analyzed data from hospitalization patients who underwent CAG and with preoperative and postoperative serum creatinine (Scr) values from January 2013 to December 2017.

**Results:**

11,943 patients were included in the study, in which the mean age was 63.01 ± 10.79 years and 8,469 (71.1 %) were male. The overall incidence of CA-AKI was 11.2 %. Compared with 2013, the incidence of CA-AKI in 2017 was significantly increased from 9.7 to 13.0 % (adjusted odds ratios [aOR], 1.38; 95 %CI, 1.13–1.68; *P*-value < 0.01, P for trend < 0.01). The temporal trends of incidence among patients of different ages and genders yielded similar findings. During a standardized follow-up of 1 year, 178 (13.7 %) CA-AKI patients died in total, which showed no obvious decreased trend in this 5 five years from 21.1 to 16.5 (adjusted hazard ratio [aHR], 0.72; 95 %CI, 0.36–1.45; *P*-value = 0.35, P for trend = 0.24).

**Conclusions:**

Our Chinese cohort showed that the incidence of CA-AKI increased significantly, while CA-AKI associated mortality showed no obvious decreased trend in the last five years. Our findings support more active measures to prevent CA-AKI and improve the prognosis of CA-AKI patients.

**Supplementary Information:**

The online version contains supplementary material available at 10.1186/s12882-021-02427-6.

## Background

Contrast-associated acute kidney injury (CA-AKI) is a common complication after coronary angiography (CAG) and interventional procedures. It is significantly associated with increased short and long-term mortality [1–4].

Several prevention strategies such as intravenous infusion, contrast media volume limiting and assessments for the risk of CA-AKI were recommended to prevent CA-AKI [5–7]. These measures were confirmed effective in the meta-analysis and have been widely accepted and implemented in the last few years [8, 9].

However, it is unclear whether the incidence and mortality of CA-AKI have changed over the last five years. The study aims to determine the incidence and prognosis of CA-AKI in a large Chinese population who underwent CAG from 2013 to 2017.

## Method

### Study design and population

Thivs retrospective study was conducted at Guangdong Provincial People’s Hospital in China (ClinicalTrials.gov NCT04407936). In total, 11,943 patients undergoing CAG and with preoperative and postoperative Scr values from January 2013 to December 2017 were included in this study.

The study was approved by the Guangdong Provincial People’s Hospital ethics committee and the study was performed according to the declaration of Helsinki.

### Baseline data collection

Data were extracted from the electronic Clinical Management System (CMS) of the Guangdong Provincial People’s Hospital from January 2013 to December 2017. The collected baseline information included demographic characteristics, coexisting conditions, laboratory examinations and medications at discharge. Senior cardiologists were responsible for the data quality control and regular database check. CAG or percutaneous coronary intervention (PCI) was performed following standard clinical practice guidelines [6, 10, 11].

### Clinical definition

The estimated glomerular filtration rate (eGFR) was calculated by the Modification of Diet in Renal Disease (MDRD) formula [12]. Chronic kidney disease (CKD) was defined as eGFR < 60 mL/min/1.73 mm^2^. Coronary artery disease (CAD), Diabetes mellitus (DM) and hypertension were identified by the 10th Revision Codes of the International Classification of Diseases (ICD-10; I20.xx–I25.xx, I50.00001 and I91.40001, see Supplemental Table S1). Congestive heart failure (CHF) was defined as New York Heart Association (NYHA) class > 2 or Killip class > 1.

### Study outcome

The primary endpoint of this study was CA-AKI which is defined as a serum Scr elevation ≥ 50 % or 0.3 mg/dL from baseline within the first 48 to 72 h after the procedure [13]. The secondary endpoint was all-cause mortality in 1 year for patients with CA-AKI. Incident events were defined as the first event occurring between the date of enrollment and the end of follow-up of December 31, 2017. All participants were followed up by office visits or telephone interviews at 1 month, 6 months and 1 year after enrollment. Follow-up data was monitored and recorded by trained nurses through outpatient interviews and telephone.

### Statistical Analysis

Patients were divided into 5 groups according to admission year. Descriptive statistics were reported as mean SD, median (interquartile range [IQR]), or number and percentage when appropriate. The chi-square test was used to compare differences between categorical variables. The differences between the 5 groups were analyzed with one-way analysis of variance (ANOVA). Pearson chi-squared tests were used to analyze the categorical data.

We calculated the annual incidence of CA-AKI from 2013 to 2017. The mortality (per 1000 persons per year) during the 1-year follow-up period was used to assess the temporal trends of CA-AKI patients’ prognosis. We used univariate and multivariate logistic regression analysis to estimate the odds ratio of CA-AKI between 2013 and 2017. Unadjusted and multivariable Cox proportional hazard regression analyses were used to explore the mortality temporal trends between 2013 and 2017. Model 1 and Model 4 were unadjusted; Model 2 and Model 5 were adjusted for age and gender; Model 3 was adjusted for age, gender, CMV, PCI, CHF and CKD; Model 6 was adjusted for multivariate variables including demographic (age, gender), medical history and procedural information (PCI, CHF, HT, DM and CKD) and medicine (ACEI/ARB, Beta-blocker). We graphically displayed unadjusted and adjusted odds ratios, hazard ratios and 95 % confidence intervals for AKI over time.

Besides, we observed the trends of incidence and mortality for CA-AKI in different age (≥ 65 and < 65 years old) and gender (male and female) subgroups. All data analyses were performed using R version 3.6.3 (R Core Team, Vienna, Austria). All P values < 0.05 were considered to represent statistical significance.

## Result

### Clinical characteristics

A total of 11,943 patients who underwent CAG were included in the study from January 2013 to December 2017. The baseline clinical characteristics of the patients were showed in Table 1. Overall, the mean age was 63.01 ± 10.79 years, and 8,469 (71.1 %) were male.

**Table 1:** Characteristics of 11,943 patients undergoing coronary angiography

Characteristic	Overall	2013	2014	2015	2016	2017	*P*-value
	(*N* = 11,943)	(*N* = 2,459)	(*N* = 2,578)	(*N* = 2,824)	(*N* = 1,960)	(*N* = 2,122)	
**Demographic**							
Age, years	63.01(10.79)	63.23(10.81)	63.24(10.93)	62.42(10.75)	63.51(10.70)	62.77(10.68)	0.003
Age ≥ 65(%)	5343 (44.7)	1116 (45.4)	1172 (45.5)	1222 (43.3)	917 (46.8)	916 (43.2)	0.068
Female, n (%)	3447 (28.9)	712 (29.0)	743 (28.8)	834 (29.5)	503 (25.7)	655 (30.9)	0.006
**Medical history**							
AMI, n (%)	2487 (20.8)	487 (19.8)	507 (19.7)	606 (21.5)	469 (23.9)	418 (19.7)	0.002
CHF, n (%)	2575 (32.6)	505 (32.7)	524 (29.2)	606 (29.7)	430 (36.3)	510 (38.5)	< 0.001
HT, n (%)	6363 (53.3)	1327 (54.0)	1426 (55.3)	1459 (51.7)	1095 (55.9)	1056 (49.8)	< 0.001
DM, n (%)	3203 (26.8)	650 (26.4)	685 (26.6)	731 (25.9)	573 (29.2)	564 (26.6)	0.116
AF, n (%)	1472 (12.3)	246 (10.0)	259 (10.0)	341 (12.1)	235 (12.0)	391 (18.4)	< 0.001
Pre-MI, n (%)	683 (5.7)	136 (5.5)	150 (5.8)	181 (6.4)	114 (5.8)	102 (4.8)	0.198
PCI, n (%)	5486 (45.9)	1330 (54.1)	1381 (53.6)	1356 (48.0)	806 (41.1)	613 (28.9)	< 0.001
CKD, n (%)	3236 (27.1)	639 (26.0)	619 (24.0)	700 (24.8)	655 (33.4)	623 (29.4)	< 0.001
**Laboratory test**							
eGFR, ml/min/1.73 m^2^	75.40 (30.25)	76.83 (39.40)	77.84 (27.34)	77.12 (27.15)	72.36 (29.26)	71.31 (25.21)	< 0.001
WBC,10^9^/L	8.05 (2.80)	8.16 (2.90)	8.01 (2.68)	8.07 (2.75)	8.11 (2.82)	7.90 (2.87)	0.021
HGB, g/L	130.41 (18.45)	131.37 (18.09)	130.20 (18.30)	131.10 (17.65)	129.84 (19.17)	129.15 (19.27)	< 0.001
CHOL, mmol/L	4.50 (1.20)	4.54 (1.18)	4.49 (1.20)	4.54 (1.17)	4.48 (1.21)	4.45 (1.27)	0.061
TRIG, mmol/L	1.65 (1.14)	1.65 (1.10)	1.59 (1.11)	1.68 (1.18)	1.69 (1.14)	1.64 (1.14)	0.032
LDLC, mmol/L	2.83 (0.98)	2.85 (1.03)	2.71 (1.00)	2.78 (0.97)	2.89 (0.91)	2.95 (0.94)	< 0.001
HDLC, mmol/L	1.00 (0.26)	1.00 (0.26)	0.99 (0.26)	1.01 (0.28)	0.98 (0.25)	1.01 (0.27)	< 0.001
HbA1c, %	6.42 (1.33)	6.52 (1.35)	6.40 (1.29)	6.33 (1.27)	6.50 (1.43)	6.39 (1.37)	< 0.001
Pre-scr, mmol/l	106.59 (89.82)	102.65 (78.24)	100.34 (72.36)	103.28 (89.45)	116.41 (99.48)	114.10 (109.32)	< 0.001
Scr-peak, mmol/l	110.09 (86.10)	105.87 (77.78)	104.75 (75.60)	108.19 (81.43)	116.95 (98.22)	117.62 (99.45)	< 0.001
CMV, ml	142.26 (97.69)	149.37 (102.36)	140.70 (92.40)	134.79 (94.34)	149.08 (103.06)	139.50 (97.44)	< 0.001
Contrast medium, n (%)							
Iopromide	4468 (40.6)	1067 (43.4)	873 (33.9)	1073 (39.9)	724 (44.2)	731 (44.2)	< 0.001
Iopamidol	5920 (53.8)	1384 (56.3)	1554 (60.3)	1356 (50.5)	781 (47.7)	845 (51.2)	< 0.001
Iodixanol	1541 (14.0)	261 (10.6)	425 (16.5)	431 (16.0)	245 (15.0)	179 (10.8)	< 0.001
Ioversol	319 (2.9)	2 (0.1)	33 (1.3)	104 (3.9)	86 (5.3)	94 (5.7)	< 0.001
Iohexol	147 (1.3)	62 (2.5)	24 (0.9)	23 (0.9)	15 (0.9)	23 (1.4)	< 0.001
**Medication**							
ACEI/ARB, n (%)	6749 (58.8)	1466 (62.4)	1472 (59.5)	1563 (57.6)	1129 (59.8)	1119 (54.5)	< 0.001
Beta-blocker, n (%)	7245 (63.0)	1419 (60.0)	1519 (61.4)	1672 (61.6)	1239 (65.6)	1396 (68.0)	< 0.001
Statin, n (%)	8801 (76.6)	1868 (79.0)	1958 (79.2)	2042 (75.2)	1490 (78.9)	1443 (70.3)	< 0.001
Dialysis, n (%)	393 (3.3)	71 (2.9)	65 (2.5)	82 (2.9)	92 (4.7)	83 (3.9)	< 0.001
Discharge status^a^, n (%)	197 (1.6)	44 (1.8)	37 (1.4)	42 (1.5)	29 (1.5)	45 (2.1)	< 0.001

Abbreviation: *AMI* acute myocardial infarction; *CHF *congestive heart failure; *HT* hypertensive; *DM* diabetes mellitus; *AF* atrial fibrillation; *Pre-MI* Previous myocardial infarction; *PCI * percutaneous coronary intervention; *eGFR* estimated glomerular filtration rate; *WBC* white blood cell; *HGB* hemoglobin; *CHOL* total cholesterol; *TRIG* triglyceride; *LDL-C* low-density lipoprotein cholesterol; *HDL-C *high-density lipoprotein cholesterol; *HbA1c* hemoglobin A1c; *Pre-scr* Preoperative serum creatinine; *Peak-scr* Postoperative peak serum creatinine; *CMV* Contrast medium volume; *ACEI/ARB* angiotensin-converting enzyme inhibitor/angiotensin receptor blocker

^a^In-hospital death /Automatic discharge

Among patients undergoing coronary angiography, 5,846 (45.9 %) patients underwent PCI treatment, CKD in 27.1 % of the patients, and DM in 26.8 %. In these five years, the patients diagnosed with CHF increased 5.9 % (from 32.6 to 38.5 %) and CKD increased 3.3 % (from 27.1 to 29.4 %). The prevalence of HT has decreased by 3.5 % (from 53.3 to 49.8 %) while there was no significant change in the prevalence of DM (from 26.8 to 26.6 %).

### Trends in the incidence of CA-AKI

**Fig. 1:**
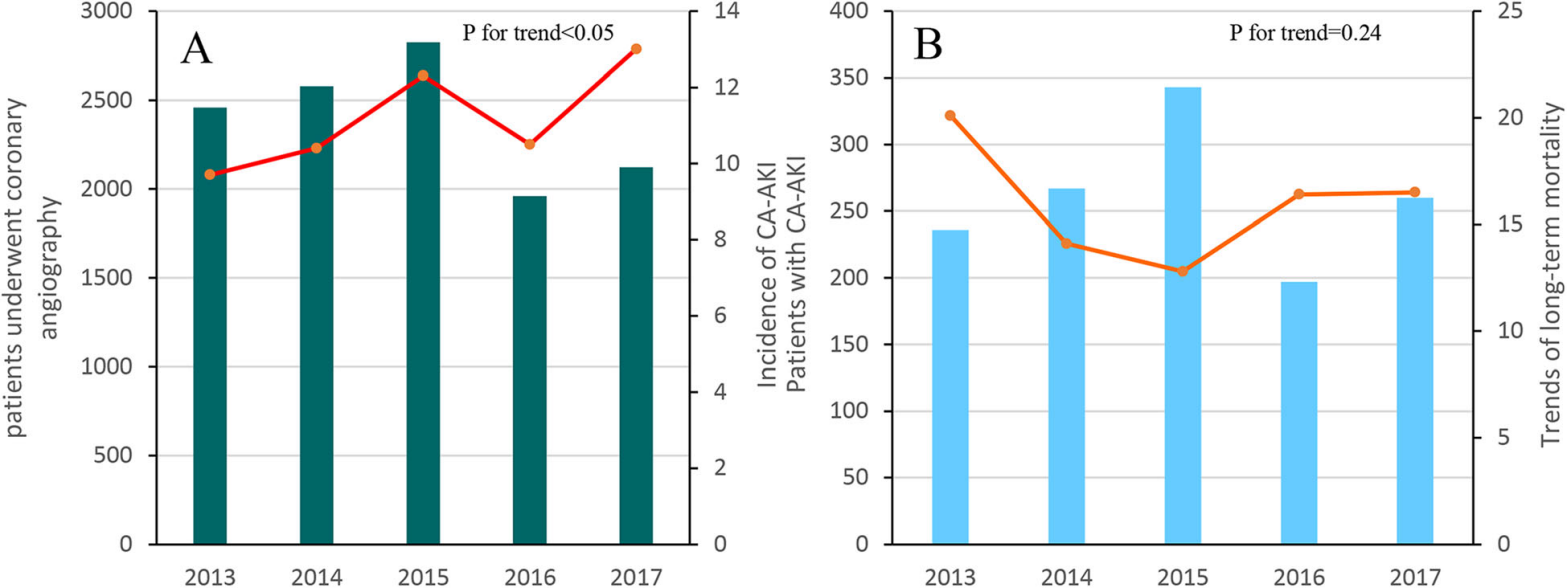
Trends in incidence and long-term mortality of CA-AKI Trends in incidence of CA-AKI among patients underwent coronary angiography in China between 2013 and 2017 From 2013 to 2017, the incidence of CA-AKI was significantly increased from 9.7–13.0 % (adjusted odds ratios [aOR], 1.38; 95 %CI, 1.13–1.68; *P* value < 0.01, *P* for trend < 0.01) Trends of long-term mortality during 1-year follow-up period among CA-AKI patients in China between 2013 and 2017 From 2013 to 2017, the long-term mortality during 1-year follow-up period showed no obvious variation trend from 21.1 to 16.5 per 1,000 person-years (adjusted hazard ratio [aHR], 0.72; 95 %CI, 0.36–1.45; *P* value = 0.35, P for trend = 0.24)

From January 2013 to December 2017, 1335 (11.2 %) patients developed with CA-AKI. Compared with 2013, the incidence of CA-AKI in 2017 was significantly increased from 9.7 to 13.0 % (adjusted odds ratios [aOR], 1.38; 95 %CI, 1.13–1.68; *P* value < 0.01, P for trend < 0.01). (Fig. 1; Table 2).

**Table 2:** Odds Ratios for prevalence and Hazard Ratios for Mortality between 2013 and 2017

**Years**	**Odds Ratios**	***P*-value**	**P for trend**
Model 1			
2013	1(reference)	-	<0.01
2014	1.09(0.90-1.31)	0.38	
2015	1.31(1.10-1.56)	<0.01	
2016	1.14(0.93-1.39)	0.2	
2017	1.45(1.20-1.75)	<0.01	
Model 2			
2013	1(reference)	-	<0.01
2014	1.09(0.90-1.31)	0.38	
2015	1.31(1.10-1.56)	<0.01	
2016	1.15(0.94-1.41)	0.17	
2017	1.48(1.20-1.75)	<0.01	
Model 3			
2013		-	<0.01
2014	1.09(0.91-1.32)	0.35	
2015	1.32(1.10-1.58)	<0.01	
2016	1.06(0.86-1.31)	0.59	
2017	1.38(1.13-1.68)	<0.01	
**Years**	**Hazard Ratios**	***P*** **value**	**P for trend**
Model 4			
2013	1(reference)	-	0.52
2014	0.71(0.45-1.12)	0.14	
2015	0.65(0.42-1.01)	0.05	
2016	0.82(0.50-1.32)	0.4	
2017	0.80(0.51-1.26)	0.34	
Model 5			
2013	1(reference)	-	0.42
2014	0.70(0.44-1.10)	0.12	
2015	0.66(0.42-1.02)	0.06	
2016	0.81(0.50-1.31)	0.4	
2017	0.76(0.48-1.19)	0.23	
Model 6			
2013		-	0.24
2014	0.77(0.39-1.52)	0.45	
2015	0.56(0.28-1.13)	0.1	
2016	0.52(0.22-1.23)	0.13	
2017	0.72(0.36-1.45)	0.35	

Model 1 and Model 4: unadjusted

Model 2 and Model 5: adjusted for age and gender

Model 3: adjusted for multivariate variables (age, gender, CMV, PCI, CHF and CKD)

Model 6: adjusted for multivariate variables (age, gender, PCI, CKD, HT, DM, CHF, ACEI/ARB and Beta-blocker)

### Trends of 1-year all-cause mortality among CA-AKI patients

During a follow-up of 1 year, 178 (13.7 %) CA-AKI patients died in total. Our data showed no obvious variation trend in this 5 five years, from 21.1 to 16.5 per 1,000 person-years (adjusted hazard ratio [aHR], 0.72; 95 %CI, 0.36–1.45; *P*-value = 0.35, P for trend = 0.24) (Fig. 1; Table 2).

### Subgroups analyses

**Fig. 2:**
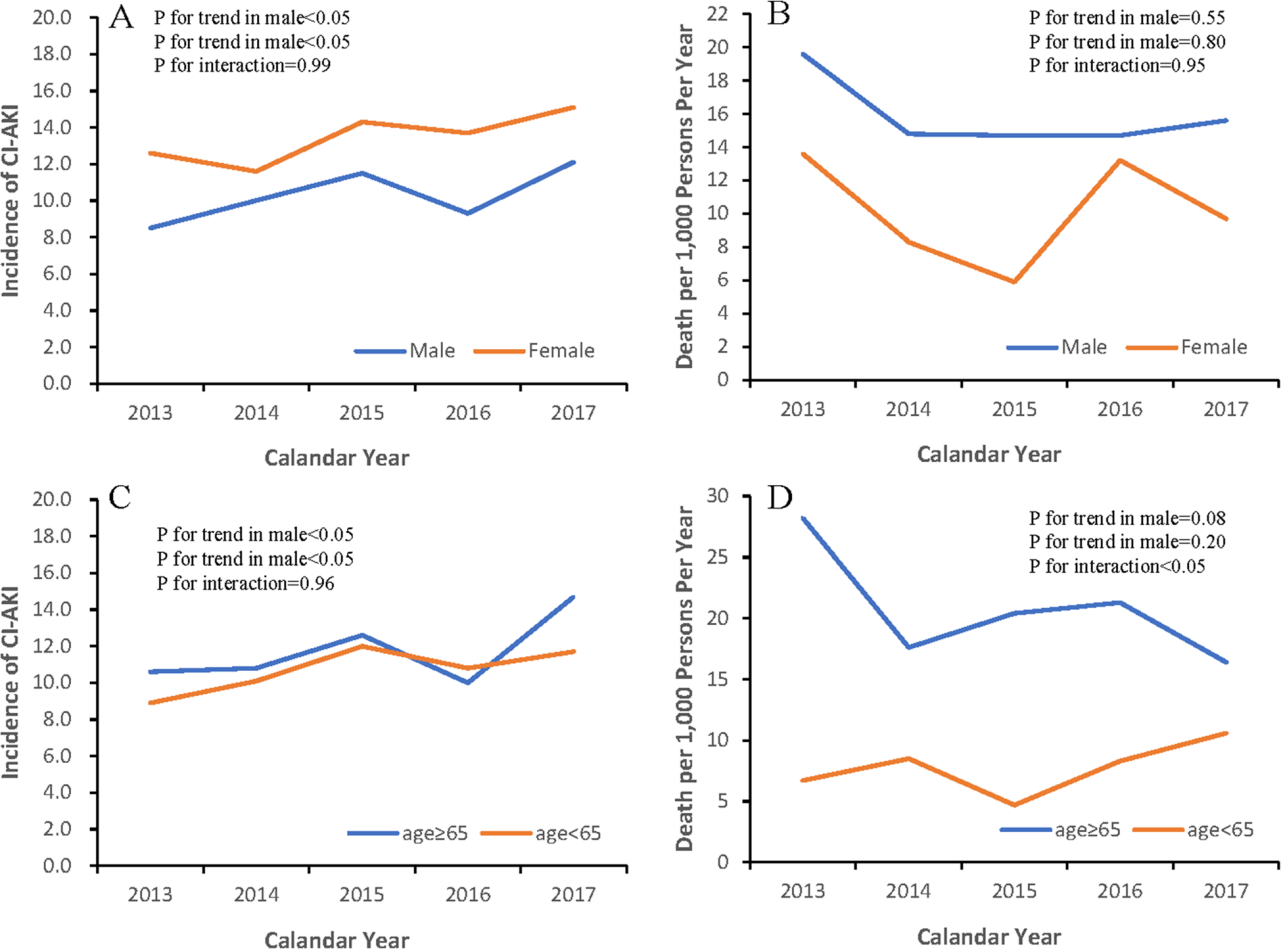
Trends in incidence and long-term mortality of 4 different Subgroups between 2013 and 2017 **A.** Trends in incidence of CA-AKI among male (P for trend<0.01) and female (P for trend = 0.03); **B.** Trends of 1-year mortality per 1,000 person-year among male (P for trend = 0.55) and female (P for trend = 0.80); **C.** Trends in incidence of CA-AKI among < 65 years group (*P* for trend = 0.01) and ≥ 65 years group (P for trend<0.01); **D.** Trends of 1-year mortality per 1,000 person-year among < 65 years group (P for trend = 0.08) and ≥ 65 years group (P for trend = 0.20)

Eight hundred seventy-two (10.3 %) patients who complicated with CA-AKI were men whereas 463 (13.4 %) were women. The incidence of CA-AKI showed an upward trend in both groups (male: 8.5 to 12.1 per 1,000 person-year, P for trend < 0.01; female:12.6 to 15.1 per 1,000 person-year, P for trend = 0.03, P for interaction = 0.99, see Fig. 2 A).

There were 134 (15.2 %) all-cause death in men and 44 (9.8 %) in women during 1 year among patients with CA-AKI. On the whole, the mortality of both groups showed a downward trend over 5 years (male: 19.6 to 15.6 per 1,000 person-year, P for trend = 0.55; female: 13.6 to 9.7 per 1,000 person-year, P for trend = 0.80, P for interaction = 0.95, see Fig. 2B).

From 2013 to 2017, 626 (11.7 %) patients developed CA-AKI in patients older than 65 years and 709 (10.7 %) in patients age < 65 years. The risk of CA-AKI in the two age groups showed an upward trend (male: 10.6 to 14.7 per 1,000 person-year, P for trend = 0.01; female: 8.9 to 11.7 per 1,000 person-year, P for trend<0.01, P for interaction = 0.96, see Fig. 2 C).

All-cause death in CA-AKI patients older than 65 years was 126 (20.6 %), and 52 (7.5 %) in patients age < 65 years. On the other hand, the mortality showed a downward trend in patients older than 65 years while showed an upward trend in patients age < 65 years (≥ 65 group, 28.2 to 16.4 per 1,000 person-year, P for trend = 0.08; < 65 group, 6.7 to 10.6 per 1,000 person-year, P for trend = 0.20; P for interaction < 0.05, see Fig. 2D).

## Discussion

To the best of our knowledge, this is the first study to explore the trends in incidence and long-term mortality of CA-AKI in the last five years. Our data suggests that the incidence of CA-AKI was 11.2 % and showed an upward trend from 2013 to 2017. Meanwhile, the findings indicate that CA-AKI associated mortality was 13.7 % and was not significantly decreased.

In our cohort, the incidence of CA-AKI was 9.7–12 % between 2013 and 2017, which was similar to the previous study regarding patients undergoing selected or emergent procedures. The incidence of CA-AKI has been reported to approximately range from 7.0 to 22.7 % [14–16]. Direct and indirect effects, as well as hemodynamic perturbations, were considered to be the main mechanisms of kidney injury caused by contrast agents. Direct mechanisms of kidney injury from exposure to contrast agents were considered to be due to nephrotoxic effects on the tubular epithelial cells, leading to the loss of cell function, apoptosis, and eventually, necrosis. The indirect mechanisms were related to ischemic damage caused by vasomotor changes mediated by vasoactive substances such as endothelin, nitric oxide and prostaglandin [3].

Our study indicated that the incidence of CA-AKI has showed an upward trend in recent years. Khera et al. found that the incidence of AKI manifested an upward trend in patients ≥ 75 years with acute myocardial infarction and undergoing primary percutaneous coronary intervention treatment from 2002 to 2010 [15]. Although that study just focused on the acute myocardial infarction subgroup among CAD populations, it has similar trends with our study.

The dose of contrast agent showed a downward trend in the last five years, while the incidence of CA-AKI showed an upward trend. There were several possible explanations for the trends towards the increased incidence of CA-AKI. First, clinicians have paid more attention to CA-AKI in recent years. Risk assessment and hydration were recommended and were proved to be effective preventive measures for CA-AKI [7, 16–18]. Increased attention to CA-AKI has resulted in more patients being evaluated, such as post-operative testing for Scr. In the past, some patients may develop CI-AKI, but postoperative creatinine was not detected, which made some patients complicated with CA-AKI be ignored. Physicians would take more preventive or curative treatments for CA-AKI among high-risk patients, which may decrease the CA-AKI associated mortality. Second, Mehran et al. indicated that CHF and CKD were the risk factors for CA-AKI [18]. In this large population cohort, the proportion of CHF, anemia and CKD were showed obvious trends of escalation, and the risk of CA-AKI among CAG patients increased.

We found that in patients of different ages and genders, the incidence of CA-AKI has shown an upward trend in the last five years (P-value for interaction > 0.05). But in patients of different ages, CA-AKI associated mortality shows different trends. The mortality showed a downward trend in patients older than 65 years while showed an upward trend in patients age < 65 years (P for interaction < 0.05). The possible reason is that in clinical practice, elderly patients are more likely to get attention after they develop CA-AKI. Doctors will take a variety of methods to treat this disease including intravenous hydration, sodium bicarbonate, acetylcysteine, and renal replacement therapy. In young patients, the slight decline in renal function after CAG is often ignored without further renal function monitoring and treatment, which leads to long-term kidney damage, even death. This reminds us that we should pay enough attention to CA-AKI, regardless of whether it is in elderly or young patients.

Many preventive measures have been proposed to prevent CA-AKI. Hydration has proven to be a very important and effective measure, which was recommended in the 2013 and 2014 clinical guidelines [19, 20]. Most of the patients included in the study received routine intravenous hydration pre- and post-CAG according to routine clinical practice. Despite receiving regular hydration, we find the incidence and mortality of CA-AKI have not been improved very well during the past five years. Due to the lack of data on the volume and the rate of hydration, we cannot further analyze the impact of hydration on CA-AKI. This is worthy of further exploration in future research.

We found that patients who underwent CAG tended to have renal function decline even dialysis. This is consistent with the findings of James MT et al. [21]. But in our cohort, patients who underwent CAG were more likely to have dialysis (3.3 %) after the procedure than in previous studies which the rate of dialysis range from 1.0 to 2.7 % [17, 22]. Compared with these studies, the patients in our study had worse baseline renal function and more complications. At the same time, these studies are all about venous hydration, the patients included in the study have received different schemes of venous hydration, which can help to reduce the risk of postoperative dialysis. Therefore, routine venous hydration and risk assessment are helpful to reduce the risk of postoperative dialysis and CA-AKI for patients undergoing CAG.

Our data suggested that CA-AKI is still a serious complication after intervention treatment. It is necessary to strengthen the prevention treatments among patients at high risk of CA-AKI, especially in CKD, elder and heart failure populations. We should strengthen the mechanism research of CA-AKI to explore more effective strategies and agents for the prevention of CA-AKI among patients undergoing CAG.

## Limitation

Our study has some limitations. First, the cohort was conducted in a single center located in south China. However, Guangdong Provincial People’s Hospital represents the high-level unit especially in the cardiovascular direction, hence our study involved abundant patients from different regions, and most of the patients complicated with different risk factors, which made the results more generalizable. Second, our cohort only included people who underwent CAG and lacked information about people who were exposed to intravenous contrast agents. Whereas the incidence of CA-AKI in CAG is significantly higher than that of intravenous contrast agents. Therefore, we should pay more attention to patients undergoing CAG. Third, we only used serum Scr elevation ≥ 25 % or 0.5 mg/dL from baseline within the first 48 to 72 h after the procedure to define CA-AKI. However, this definition has reasonable sensitivity and is widely recommended and used internationally. Fourth, this dataset lack off several information about the stage of AKI, shock events, emergent/urgent CAG or elective CAG, and the volume intravenous hydration, but we have considered several relevant variables and shown in Table 1, such as acute myocardial infarction (AMI).

## Conclusions

This study demonstrated that the incidence of CA-AKI was significantly increased, while the CA-AKI associated mortality showed no obvious decreased trend in the last five years. These findings highlighted that CA-AKI is still an important public health problem in China. Further research would need to explore more strategies to decrease the incidence of CA-AKI and improve the prognosis of CA-AKI patients.

## Supplementary Information

**Additional file 1.**

## Data Availability

The datasets used and/or analyzed during the current study are available from the corresponding author on reasonable request.

## Abbreviations

ANOVA: One-way analysis of variance
CA-AKI: Contrast-associated acute kidney injury
CAD: Coronary artery disease
CAG: coronary angiography
CHF: Congestive heart failure
CKD: Chronic kidney disease
CMS: Clinical Management System
DM: Diabetes mellitus
eGFR: Estimated glomerular filtration rate
MDRD: Modification of Diet in Renal Disease
NYHA: New York Heart Association
PCI: Percutaneous coronary intervention
